# Breaking Bad News to Learners: How Well Does the SPIKES Clinical Model Translate?

**DOI:** 10.5334/pme.1521

**Published:** 2024-12-27

**Authors:** Lynnea M. Mills, Olle ten Cate, Christy Boscardin, Patricia S. O’Sullivan

**Affiliations:** 1 University of California, San Francisco, US; 2 University Medical Center Utrecht, NL

## Abstract

When health professions learners do not meet standards on assessments, educators need to share this information with the learners and determine next steps to improve their performance. Those conversations can be difficult, and educators may lack confidence or skill in holding them. For clinician-educators with experience sharing challenging news with patients, using an analogy from clinical settings may help with these conversations in the education context. One common model in the clinical setting for ‘breaking bad news’ to patients is SPIKES: Set-up, Perception, Invitation, Knowledge, Emotion, and Summary/Strategy. The authors reviewed evidence in the education setting, particularly from the remediation literature, to consider how the SPIKES model might translate from clinical settings to those in which educators must share ‘bad news’ with learners about their academic performance. Based on available guidelines and evidence, the authors advocate that the SPIKES model can serve as a useful framework to help educators incorporate, by way of analogy, key components into these conversations, and increase the likelihood of successful outcomes.

Many health professions educators will, at some point, need to disclose to learners that they have not met academic performance standards. Learners may fail tests, fall short of clinical competencies in assessments, or demonstrate behaviors that do not meet professional standards. Then, education leaders must talk with the learners about their performance and discuss next steps to remedy the situation, where applicable. In health professions education, the need for these conversations is common [1], but many educators feel nervous engaging in these discussions; some even avoid them altogether [2]. The avoidance of these conversations can lead to a ‘failure to fail’ phenomenon in clinical education [3]. Sharing honest information with learners about assessed performance is a professional obligation for educators, but many lack support, resources, or frameworks to guide these conversations.

## The patient care – education analogy in breaking bad news

Clinician-educators who trained first as clinicians may find value in extending or analogizing clinical models or frameworks into education settings. For example, ‘precision education’ [4], ‘diagnosing the learner’ [5], ‘high-value education,’ [6] and ‘educational alliance’ [7] are health professions education concepts that draw on language common to clinical settings. The use of these clinical terms in education contexts may help clinician-educators to draw parallels to the educational meaning and more easily implement the relevant skills. Analogies are powerful tools that enable processing and learning of a less familiar entity by using a more familiar model to support conceptual mapping and transfer [8]. Using analogies from the clinical space in education contexts can help educators remember and use key components of important conversations with learners [9].

For these reasons, we suspected that analogizing from patient care models might feel useful to educators who need to disclose suboptimal performance assessments to learners but feel uncertain how to do so. In the patient care setting, sharing challenging information with patients about their health is often termed ‘breaking bad news’ (BBN).^1^ Communication and palliative care scholars have explored BBN extensively and have developed models to help guide clinicians. To determine if and how patient care models for BBN might work in education settings, we first identified in the literature areas where the process of conveying this news is suboptimal. For example, some struggling learners find that ‘assessment failure’ conversations focus solely on improving performance, without factoring in their emotions or challenges in their outside lives [11]. Upon hearing the news, learners may experience strong negative emotions that hinder their ability to engage with ongoing skill development [12]. They may also find that hearing the news without detailed information about next steps produces stress and anxiety [13]. If institutions have not normalized failure for these learners, the learners may feel isolated and doubt their adequacy for a career in medicine; news-givers may not appreciate the gravity of these experiences for learners [13]. These findings held parallels to patient care settings and led us to believe that using guidance from the patient care literature, particularly where it emphasizes attending to emotion, could help educators in these conversations with learners. We consider the translation of clinical models of BBN to the education context by utilizing the literature on failure and remediation for health professions learners to provide specific guidance on when and how the patient care models translate.

‘Bad news’ in clinical contexts is defined as any information that changes patients’ expectations in a negative way [14]. We sought out the most common model for breaking bad news in clinical settings, in hopes that this would be familiar to, or at least easily understood by, many clinician-educators. A recent systematic review of evidence around these conversations identified multiple models [15], but most (e.g., “ACBDE” model) are studied infrequently in the literature. We sought a well-researched model with a strong evidence base for clinical success, and which emphasized attending to individual needs and emotions. We found these criteria were achieved in the SPIKES model, developed by Baile and colleagues in the late 1990s in the oncology setting [16]. The model was based on research on clinician needs in sharing bad news, as well as extensive prior research on best practices for different components of the conversation. SPIKES stands for Set-up, Perception, Invitation, Knowledge, Emotion, and Strategize or Summarize [16]. Clinicians subsequently expanded this model widely into all clinical domains where providers share information that negatively changes patients’ expectations for the future; it is now commonly taught for breaking bad news across clinical settings [17]. Multiple studies and a systemic review have shown the SPIKES model to improve clinician self-confidence in BBN conversations, and clinicians who are taught the model have improved outcomes [17]. In educational settings there is no recommended protocol for conveying bad news. We therefore explored to what extent SPIKES might serve educational BBN conversations.

## SPIKES for bad news conversations in education contexts?

Breaking down each component of this model can help reveal how well the model translates into the education setting. (See Table 1 for a summary of this translation to education.)

**Table 1:** Translation of the SPIKES protocol for breaking bad news from patient care settings to education settings.


FRAMEWORK COMPONENT	DESCRIPTION IN THE PATIENT CARE BBN CONVERSATION PROTOCOL	RECOMMENDATION FOR USE IN BBN CONVERSATIONS WITH LEARNERS

**S**et-up	Gather all necessary information (e.g., test results) to inform the discussion. Identify a space and time for the conversation that will maximize patient comfort and privacy.	Prepare by considering as much as possible what information the learner may need to understand the situation, and what might help them feel most comfortable as they receive the news. Recognize these will vary across learners.

**P**erception	Elicit the patient’s understanding of their condition and what it may mean for their future health.	Ask the learner for their perception of the assessment and their overall performance.

**I**nvitation	Allow patients to share preferences around receiving the news, including the level of detail they’d like to know and who else to involve.	Though learners may have fewer options than patients (i.e., learners cannot defer all decision-making to the educator the way a patient could to a provider or surrogate), maximizing learners’ ability to share their preferences for the conversation can advance learner autonomy.

**K**nowledge	Share the information in clear terms without jargon or euphemisms. Some experts recommend preceding the actual news with a ‘warning shot’ so patients feel more prepared.	Emphasize clarity of language. Currently cannot recommend for or against avoidance of euphemisms.

**E**motion	Expect, make space for, and validate/respond to patients’ emotions with empathy.	Educators should expect strong emotions from learners, including concerns about professional identity and fairness. Educators should expect they may experience strong emotions themselves.

**S**ummarize/**S**trategize	Summarize the discussion, ideally in a way patients can reference later. Discuss next steps and work together to develop a plan. Indicate ongoing involvement and support for the patient.	Learners are often well-equipped to engage in shared decision-making on next steps.


### Set-up

In the patient care application of BBN, set-up encompasses multiple types of preparation. The first is for the clinician to gather all relevant information (diagnostic and prognostic information, treatment options, etc.). The second is to recognize and prepare for the impact the information could have on the patient by creating a supportive space in which to learn the information: a private, comfortable setting with the right people present [18]. Additionally, clinicians need to anticipate and reflect on the impact that these conversations will have on them as bearers of the news [19].

In the context of working with learners, these elements of preparation also apply. When preparing to bring in the relevant information, educators often possess the results of trainee assessments, in the form of test scores, supervisor comments, etc. Learners may often fear that an academic failure will have significant impact on their career trajectory [13]. Therefore, educators may want to anticipate (sometimes unasked) learner questions about what this news means for their overall educational trajectory (‘prognosis’). Educators also need to consider carefully the setting in which learners receive the news of their performance assessment. General advice in the patient care domain is to share bad news with patients via direct, in-person conversations if possible [18]. Although learners vary in their preferences, many feel depersonalized when receiving this information through indirect means such as form-letter emails or class grade postings online [13]. This indicates the importance of tailoring the conversation to the individual as much as possible. Having a direct conversation may help learners feel that their educators truly understand the impact of the news on learners. It may feel similar to patient conversations in this way.

Educators are likely to experience strong emotions when delivering bad news to learners, just as clinicians do with their patients. Those delivering the news about student assessment have reported discomfort with the conversations [2]. However, we do not have extensive data about how educators’ experiences may differ from those of clinicians in patient care BBN conversations, and whether reflective processes outlined in the literature for clinicians (e.g., the ‘Preparatory SPIKES’ [20]) are applicable. While it’s unclear at this stage what specific practices to recommend, some form of personal reflection and emotional preparation is likely important for educators delivering bad news, given the potential impact on them.

### Perception

The patient communication literature frequently recommends beginning challenging conversations by eliciting the patient’s perspective: what does the person already understand about their illness, and what do they expect to happen [18]? This approach allows the clinician to assess where the patient is, frame the discussion around the patient’s current understanding, and build more quickly to a point of mutual understanding. It may also help the patient feel heard.

Eliciting the learner’s perspective and self-assessment is a key part of good feedback conversations [21] and is similarly relevant to BBN conversations with learners. The need to align the learner’s and educator’s perceptions of the situation is just as important as it is in the case of patients and clinicians. While many patients have significant agency in their healthcare, in some clinical situations it may be possible to move forward without the patient having a full understanding of the clinical situation, and some patients may even prefer not to know details. (See below, in “Invitation.”) Learners may have more difficulty making necessary improvements in their performance without an understanding of the learning goals and where they are assessed as falling short. Initiative to improve requires insight on the part of the learner [22]. Medical learners across levels are generally poor at self-assessing their skills and often require guidance to see their performance as others do [23,24]. For this reason, the initial phase of elicitation of their perspective, and eventual alignment of understanding with the educator, is particularly important for moving forward.

### Invitation

Patient communication guidelines recommend giving the patient some control over their experience of hearing the information the clinician wants to convey; the clinician should therefore wait for an ‘invitation’ to share the information in the manner best for the patient. This can range from determining who else should be present, to delaying the disclosure of the news to a more comfortable moment. Patients may decide the amount of detail that gets shared, or even avoid hearing the news themselves, instead asking the clinician to convey it to a surrogate. All of these are acceptable requests for the clinician to honor, given that patients’ psychological outcomes improve when clinicians tailor information-sharing to patients’ preferences [25].

Some options clinicians afford patients around news disclosure may also be applicable to learners, such as who else should be present to hear the news and how it can be framed to maximize understanding. Other options may not be available to learners, though. For example, educational timelines may preclude educators from being flexible about when to disclose the news. Additionally, because learners need to take an active role in their improvement plans, they do need to understand the details of the assessments. Learners cannot ‘outsource’ this knowledge to a surrogate. Hearing the details of external assessments is vital to developing action plans, particularly because learners’ self-assessments are often limited during feedback conversations about their performance [26], For these reasons, the ‘I’ in the SPIKES model takes on a different role in the education space. However, maximizing the ‘I’ when possible may help learners feel they have some autonomy in the process of learning the news, given what we know about learners’ preferences for having news tailored to their individual needs [13]. It’s reasonable to assume that a learner who is able, for example, to invite a trusted advisor to join the conversation, or to specify whether they want to receive the information in-person vs by phone, may feel more agency and empowerment in moving forward with next steps.

### Knowledge

In this portion of the BBN conversation with patients, clinicians share the news. This often involves disclosing a serious new diagnosis, conveying a poor prognosis, or advising of a change in care plan, such as the clinician leaving the practice. Many experts suggest giving a small preview of the information (sometimes termed a ‘warning shot’) to help patients prepare to hear and process difficult information [27]. This can come at the beginning of the BBN conversation or can be conveyed in a message prior to that conversation. For example, a clinician might say, ‘we got the results of your scan and I want to talk with you about some serious things we saw.’ Then they should disclose the information in a straightforward way, avoiding jargon [16,28]. Experts also recommend avoiding euphemisms and speaking frankly when discussing serious information with patients, including avoiding terms like ‘pass away’ [28].

These same guidelines can apply to BBN with learners. On a practical level, if an educator contacts a learner to set up a meeting but isn’t specific that the meeting is to discuss a substandard performance, that message may still serve as a ‘warning shot’ for learners who are not used to being called to meetings. If there was no warning embedded in the meeting set-up, it may be helpful for the educator to provide a brief verbal warning before diving in. For example, the educator could say, ‘I wanted to discuss your performance on the recent assessment.’ Although we do not have specific data about this approach for learners, this type of warning may give them opportunity to reclaim as much agency as possible, such as requesting how the news is presented to them.

While clarity of language is likely as important for learners as for patients, there are mixed data in this space. Although the patient care literature suggests avoiding euphemisms, this approach is less clear in the education space. For example, some learners have expressed preferences to move away from terms like ‘remediation’ [29] and the literature has largely pivoted to phrases that may be viewed as euphemisms, such as ‘struggling learner’ [30] or ‘learner in difficulty’ [31]. However, some learners do continue to use words like ‘fail’ and ‘remediate’ spontaneously and explicitly [13], so there is a need for further work to provide guidance on language usage in this context.

### Emotion

Recognizing and responding to patients’ emotions is a key piece of the clinical BBN conversation. While some clinicians may feel nervous or uncomfortable holding space for strong emotions in patients, numerous studies have demonstrated the importance of allowing patients the opportunity to feel and express emotions during the clinical encounter [16,18]. During BBN conversations, this often involves silence (which can convey to the patient that the clinician recognizes the gravity of the news and expects and welcomes emotion, or can allow the patient time for processing and collecting oneself), empathetic body language (facing the patient, not typing), asking open-ended questions to allow patients the opportunity to share the emotions they’re experiencing, and providing validation and empathy around those emotions [16].

Recognizing and responding to learner emotions may feel different or less familiar, particularly because of the culture in medicine that has historically avoided directly addressing clinicians’ emotions [32]. Some clinician-educators may have more experience engaging with patients’ emotions than with learners.’ Yet this component of the framework is equally important when breaking bad news for both learners and patients. Learners experiencing academic failure or underperformance may experience intense emotions. For certain types of academic challenges, learners may view the situation as carrying far more gravity than the educators are aware [13]. For example, failure of a pre-clerkship exam may not feel particularly noteworthy to an educator in a learner’s overall trajectory, but to the learner that failure may feel highly significant at that moment. Educators need to anticipate this perspective and hold space for the associated emotions during the BBN conversation.

Though learners, like patients, may have strong emotions when receiving bad news, the specific emotions they experience are likely different. With some types of bad news (e.g., diagnosis of a condition related to smoking), some patients may feel responsible, but for many types of bad news the patients are likely to attribute the issue to forces beyond their control. Learners are more likely to consider this type of bad news to be a reflection on them as individuals. Learners in this situation often feel shame [13,33] and self-doubt, and for many this represents a threat to their identity as developing physicians [13].

The emotion toward the messenger of the news may also be quite different for learners compared to patients. Though in some situations patients may assign responsibility for the bad news to their clinicians, and certain ways of disclosing exacerbate this reaction [16], there are likely many scenarios where patients attribute their situations to forces unrelated to the clinician. For learners, though, the person delivering the news about their performance is often the person who assessed that performance; learners may attribute blame to educators for what they feel to be unfair assessments or poor teaching [13] and may feel angry as a result [13]. Overall this concept of attribution of responsibility creates a level of nuance in the education setting that is different from the clinical setting. Attribution theory [34] has often been applied to the causes learners attribute to their academic failures. It distinguishes three axes: internal versus external locus of causality (‘not my fault because the exam was unfair’), (in)stability (‘I was sick that day’) and (un)controllability (‘I have never been good at this subject and will never be’), leading to learners attributing failure to lack of ability, lack of effort, task difficulty, or bad luck. All of these axes are likely present for remediating learners, which creates certain challenges that differ from those in patient care settings. And because the particular attribution learners assign for certain educational outcomes can impact their future motivations and behaviors [35], the emotions related to learners’ attribution may be especially important. Normalizing and empathizing with the learners’ emotions while also standing by the determination of the assessment may be challenging for educators to navigate but will be crucial for learner processing of the news.

Educators may feel reassured to know that the immediate emotional reactions do not always persist for learners [13]. In many cases, with the passage of time, as well as ongoing work toward their professional goals, learners change perspectives on their failure event. This often includes a decrease in the size and intensity of the emotions, as well as a greater belief that the event does not define them as clinicians [13]. When responding to immediate emotions during the BBN conversation, we would not recommend telling a learner they will likely feel differently later, but we hope this awareness may allow educators to feel less worried or guilty about perceived negative emotions they create for learners when sharing challenging news.

While some learners may not experience heavy emotions when receiving this type of news, some certainly do, and educators may not be able to predict or read the emotion the learner is experiencing. For this reason, emphasizing the ‘E’ in the SPIKES model allows educators to create space for learners to bring forward whatever their experience may be and to respond empathetically in a way that may strengthen the learner’s sense of support from and alignment with the educator.

### Summarize and Strategize

When sharing serious news with a patient, clinicians are advised to summarize the news concisely and potentially via an additional modality, such as writing, so that patients can continue to process it after the conversation [36]. Clinicians should also expect that patients may need to revisit the information in subsequent conversations to grasp it fully. This phase of the conversation is also an opportunity to strategize about next steps, including explaining treatment options and other medical decisions to be made, and working together to determine a plan.

Learners, likewise, should be offered summaries of the discussions. It’s important to summarize the information in a durable, written format for learners. This may help protect the educator in case of any necessary escalation of action in the future, but the main reason to do this is to help the learner with next steps. Many learners feel uncertain about next steps and feel an urgency about understanding the full ramifications of the underperformance and processes to remedy it [13]. Providing this information in written format for them to refer to may help alleviate some of this stress.

Strategizing around next steps is vital for learners to improve after a suboptimal performance assessment, so this step of the model translates well from clinical to educational settings. While clinical best practices around treatment plans include shared decision-making [37], patients are in many ways dependent on their clinicians for necessary components of the interventions, including referrals, prescriptions, procedures, and more. Learners confronting bad news about their performance or skills can exercise more autonomy in their next steps, and, in some settings, develop their own remediation plans [38]. In this way, the advised strategy of shared decision-making may be even more feasible in education settings than clinical settings. Some learners may be able to meet remediation goals without any outside assistance, while others need or appreciate involvement of coaches [13]. Studies indicate that coaches, or any support person, should be a third-party who does not assess the learner [39] and is not the person who delivered the bad news.

While some patients may receive news and then have minimal ability to engage in next steps that can change the outcome (e.g., a very limited prognosis), some patients receive news that can spur next steps, providing them significant agency. For example, patients who receive a new diagnosis of hyperlipidemia may then feel motivated to make healthy lifestyle changes; ultimately, then, what initially felt like ‘bad news’ may come to be viewed in a different light. Some learners may also use the initial news of their performance assessment to prompt durable and valuable changes (e.g., failing a test may lead to a learner developing better study habits). In this way, a ‘growth mindset’ [40], the belief in one’s ability to improve and grow with time and effort, is presumed to be valuable for learners who receive this type of news [41]. However, the medical education literature has not yet extensively explored how best to promote and leverage a growth mindset in the remediation context, nor demonstrated in what ways it may help. This area deserves further research. Meanwhile, explicitly asking learners to consider how they can use the news to springboard them into a better position going forward, and emphasizing long-term impacts instead of short-term gains [39], is likely a valuable first step in this stage of the conversation. Clinicians may find this similar to motivational interviewing for patients [42].

A key step for many patients who are adjusting to news is to be connected with others living the same experience. Many patients benefit from support groups with others with the same diagnoses or clinical scenarios [43]. This has not been standard practice for struggling learners, and the stigma around failure/remediation often leads to an understandable desire to maintain confidentiality. That said, learners often report benefits from being connected with others going through the same experience [13], so this may be an important area for educators to consider as they strategize about next steps after breaking the news. In this way, the model extends well from patient care to learner support.

## Implications

Talking with learners about situations in which they do not meet academic expectations will always be a part of health professions education. As we shift toward programmatic assessment, which emphasizes a greater number of formative assessments [44], summative assessments may be less surprising ‘news’ for learners. However, there will likely still be many situations in which skill in BBN is important, for multiple reasons. Even small formative assessment points may still carry great weight for some learners [45], who research indicates appreciate careful attention to the delivery of the information. Learners will vary in their perceptions, so it may be impossible to ensure that every learner interprets the formative assessments as ‘adding up’ to the same summative assessment the educator sees. Finally, even if a below-expectations outcome on a summative assessment is not unexpected for a learner, it may still feel overwhelming or highly significant, thus indicating a need to host the conversation with skill. Yet educators lack widely available guidelines or frameworks for how to engage in these discussions in a way that leverages the evidence from education and is best poised to improve learner outcomes.

The SPIKES framework provides helpful guidance for clinicians who need to share difficult news with patients, and evidence from medical education suggests it may also serve as a useful model when educators share news of underperformance with learners. While educators will need to take other important steps to maximize the success of these conversations, including considering their credibility as news-givers and the educational alliance they have with a given learner [7] (similar to the relational work clinicians do with patients), the SPIKES model can be helpful for the conversation itself. In efforts to consider appropriate clinical models for BBN in education settings, we spoke with clinicians who serve in remediation leadership roles, as well as palliative care providers who also have education leadership roles, and they all perceived that SPIKES could be useful in the education context, thus meriting further exploration.

Educators should always be cautious before translating clinical models into education settings, as doing so risks pathologizing learners and/or misapplying the models. But in this case careful review of the data in the education context indicates that translation of SPIKES is likely appropriate, if done with thought and attention to key differences. Carefully outlining the fidelity of the mapping from the well-known to lesser-known concept can avoid confusion when adopting analogies [46], so we have outlined those differences here. They include the fact that learners and patients will have different ways in which they can make requests about the conversation (“Invitation”) and different emotions they will likely experience during the conversation (“Emotion”).

Each component of the SPIKES model can apply to disclosure of bad news to learners. In fact, for some components of the model, particularly ‘emotion,’ the significant relevance and importance in the education setting lends credence to the idea of translating the model. If the mnemonic helps educators remember to engage in these key steps when sharing bad news, they will have aligned with important findings in the remediation literature and benefited their learners. While translation of the SPIKES model may be valuable for those who don’t have clinical experience with the model, it is especially relevant for those who are already familiar with using SPIKES in clinical settings. Sharing challenging news is a difficult but necessary part of many educators’ roles and we hope this model may help many feel more comfortable to engage in those interactions.

## Footnotes

The term ‘breaking bad news’ has several challenges, including the fact that the perception of the ‘badness’ and ‘newness’ of the information may vary across patients [10]—concerns which will exist when translating into the education space, too—but we use it here because of its prevalence in the literature and the lack of a widely-used substitute at this time.
